# Perceptions and acceptability of co-administered albendazole, ivermectin and azithromycin mass drug administration, among the health workforce and recipient communities in Ethiopia

**DOI:** 10.1371/journal.pntd.0011332

**Published:** 2023-10-02

**Authors:** Scott McPherson, Dereje Geleta, Getinet Tafese, Temesgen Tafese, Sinkinesh Behaksira, Hiwot Solomon, Birhanu Oljira, Hirpa Miecha, Lalisa Gemechu, Kaleab Debebe, Biruck Kebede, Teshome Gebre, Fikreab Kebede, Fikre Seife, Fentahun Tadesse, Belete Mammo, Abraham Aseffa, Anthony W. Solomon, David C. W. Mabey, Michael Marks, Endalamaw Gadisa

**Affiliations:** 1 Clinical Research Department, Faculty of Infectious and Tropical Diseases, London School of Hygiene & Tropical Medicine, London, United Kingdom; 2 RTI International, Durham, North Carolina, United States of America; 3 College of Medicine and Health sciences, Hawassa University, Hawassa, Ethiopia; 4 Armauer Hansen Research Institute, Addis Ababa, Ethiopia; 5 Disease Prevention and Control Directorate, Ministry of Health, Addis Ababa, Ethiopia; 6 Oromia regional Health Bureau, Addis Ababa, Ethiopia; 7 International Trachoma Initiative, Task Force for Global Health, Addis Ababa, Ethiopia; 8 Department of Control of Neglected Tropical Diseases, World Health Organization, Geneva, Switzerland; 9 Hospital for Tropical Diseases, University College London Hospital, London, United Kingdom; 10 Division of Infection and Immunity, University College London, London, United Kingdom; Washington University School of Medicine, UNITED STATES

## Abstract

Several neglected tropical diseases (NTDs) employ mass drug administration (MDA) as part of their control or elimination strategies. This has historically required multiple distinct campaigns, each targeting one or more NTDs, representing a strain on both the recipient communities and the local health workforce implementing the distribution. We explored perceptions and attitudes surrounding combined MDA among these two groups of stakeholders. Our qualitative study was nested within a cluster randomized non-inferiority safety trial of combined ivermectin, albendazole and azithromycin MDA. Using semi-structured question guides, we conducted 16 key informant interviews with selected individuals involved in implementing MDA within the participating district. To better understand the perceptions of recipient communities, we also conducted four focus group discussions with key community groups. Individuals were selected from both the trial arm (integrated MDA) and the control arm (standard MDA) to provide a means of comparison and discussion. All interviews and focus group discussions were led by fluent Afaan oromo speakers. Interviewers transcribed and later translated all discussions into English. The study team synthesized and analyzed the results via a coding framework and software. Most respondents appreciated the time and effort saved via the co-administered MDA strategy but there were some misgivings amongst community beneficiaries surrounding pill burden. Both the implementing health work force members and beneficiaries reported refusals stemming from lack of understanding around the need for the new drug regimen as well as some mistrust of government officials among the youth. The house-to-house distribution method, adopted as a COVID-19 prevention strategy, was by far preferred by all beneficiaries over central-point MDA, and may have led to greater acceptability of co-administration. Our data demonstrate that a co-administration strategy for NTDs is acceptable to both communities and health staff.

## Author summary

The strategy for several neglected tropical diseases is treatment of the whole community, referred to as mass drug administration. Normally these are delivered as separate rounds of treatment for each disease. This creates a burden for both the health workforce and the communities. As part of a larger study conducted in Ethiopia we used interviews and focus groups to explore perceptions and attitudes towards combined mass drug administration among both communities and health workers.

Both community members and health workers appreciated the time and effort saved via combined treatment although some community members were worried about the number of pills that had to be taken. Both health workers and community members said some people declined to take part due to a lack of understanding around the need for a combined drug regimen. Delivery of the drugs house-to-house, which had been adopted as a COVID-19 prevention strategy, was preferred over delivering the treatment at a central point in the community. Overall, our study showed that combined treatment of multiple neglected tropical diseases was acceptable to both communities and health workers.

## Introduction

Currently, mass drug administration (MDA) for neglected tropical disease (NTD) control and elimination takes place through several distinct campaigns, with the number of such campaigns depending on the number of diseases being targeted. If trachoma, lymphatic filariasis (LF), and onchocerciasis are all co-endemic, the first MDA round that takes place addresses onchocerciasis and LF through a height-dependent dosage of up to four pills of ivermectin and, regardless of height or age, one pill of albendazole; in some populations, diethylcarbamazine is also added. The second round of MDA takes place for trachoma at least two weeks later, involving a height-dependent dosage of up to four pills of azithromycin, a height-dependent dose of azithromycin oral suspension, or (for children aged <6 months or those unable or unwilling to take macrolides) tetracycline eye ointment (TEO).

Several studies have evaluated the safety of co-administration of these medicines as a strategy to tackle multiple NTDs in an integrated manner. A recently completed cluster randomized controlled trial (RCT) conducted in Ethiopia demonstrated that albendazole, azithromycin and ivermectin can safely be administered together in a single, combined MDA campaign [1]. These data add to existing evidence on the safety and feasibility of co-administration generated from both RCTs and cohort studies [2–7]. The acceptability of the approach has not previously been formally assessed.

Ultimate uptake of integrated MDA for NTDs will depend on the acceptability of the intervention to both intervention deliverers and recipients [8]. From the healthcare worker perspective, co-administration may present an opportunity to significantly reduce the time commitment demanded from the local health workforce. In theory this could free up the local health work force for other commitments. However, co-administration might be more complex and require additional training or supervision. It is also important to note that the COVID-19 pandemic has resulted in modifying MDA distribution in several countries from a central point distribution strategy to a house-to-house approach to encourage social distancing. It is important to consider how these two different distributions strategies impact the acceptability of co-administration.

While the workload of the health work force is an important consideration, it’s also important to consider its acceptability to those receiving the intervention. Co-administration may present an opportunity to significantly lessen the time investment required for beneficiaries to participate in MDA and reduce the indirect costs of participation. Ideally, this reduction in indirect costs would subsequently improve MDA coverage as participants would not have to choose between participating in multiple MDA rounds and their other obligations. Co-administration might alternatively result in decreased coverage if enough individuals decline to swallow the larger number of tablets offered as part of a combined MDA approach or have other safety concerns.

To assess the acceptability of the co-administration strategy from both provider and recipient perspectives, we nested a qualitative study within our recently conducted RCT in Ethiopia.

## Methods

### Ethics statement

The study was nested within the larger co-administration RCT. The study received ethical approval from the National Research Ethics Review Committee (NRERC) of Ethiopia (reference 3-10/195/2018) and the London School of Hygiene & Tropical Medicine (reference 11985). Based on low levels of literacy in the study population, permission to use verbal consent was specifically provided by the ethics committees. Study teams read the study consent form to all prospective participants and obtained verbal permission to participate in the study.

Interviewers used a digital audio recorder during the interviews. They uploaded the audio files to the RedCap account created for the study which had a specific passcode. Once uploaded, the files were deleted from the digital recorder. The two interviewers then completed transcription and translation on the computer and also uploaded these to RedCap. Each individual interviewed was anonymized via a specific code during transcription.

### Study setting

The methodology of the RCT within which this study was conducted has been described elsewhere [9]. Briefly, the study took place the two kebeles (sub-districts) Gurmicho and Alkaso, of Kofele woreda (district) in Oromia regional state. All previous rounds of standard MDA in Kofele woreda have had a coverage above 90% for trachoma and 75% for LF but coverage information was not available on an individual kebele level. Within these kebeles, communities were randomized to receive either combined MDA, consisting of a single MDA for lymphatic filariasis and trachoma delivered on the same day, or standard MDA, consisting of MDA for lymphatic filariasis and trachoma administered separately at least two weeks apart.

### Participant characteristics

A total of 49 participants were included in the study, including 16 health worker key informants and 33 participants who contributed to four focus group discussions. The focus groups averaged 8 participants each were evenly recruited from the coadministration and control arms. In study communities, Islam is the dominant religion. Farming is the main means of earning a livelihood followed by animal rearing. Administratively, villages are led by kebele leaders, generally politically appointed by the district administration. Besides kebele leaders, community elders and religious leaders play a significant role in different political and social activities in the community. The Gada is a traditional system of governance of the Oromo people built off of community experience over generations which encompasses of all of the socio-political issues within a community. The leader of the Gada is ‘Aba Gada’ or father of the Gada. Community structures like Women and Youth associations and Women health development armies in the village also contribute to various local developmental activities (For further description of community and health worker roles see: S1 Text)

### Participant selection

Participants for key informant interviews included members of the health development army and health extension workers involved in delivering MDA; religious and community leaders; and NTD focal points at woreda and zonal levels. Focus groups included both genders and ensured representation of both younger and older members of the community. Each focus group consisted of eight to ten people, each with participants evenly divided from both the trial arm and the control arm (S1 Table). In previous years, all participants had taken part in standard MDA.

For individual participant selection, the study team engaged with the local leadership and health workforce with some specific criteria. The study team stressed that those selected should have lived in the community for at least a year and should be open to engaging in discussion. The study team further stressed that participants should live in different *gotes* (sub-districts) where the study took place.

The kebele leader recommended the religious and community leaders interviewed as well as all of the males in the focus groups. The most senior Health Extension Worker, a position predominantly held by a woman, made the recommendations for the members of the Health Development Army and for the females in the focus groups.

### Interviews

The study team conducted semi-structured in-depth interviews with health care workers and focus group discussions (FGD) with members of the community using a semi-structured question guide. This interview guide was created specifically for this study and had not been previously tested. A native Afaan oromoo speaker conducted all key informant interviews (KII) and led the focus group discussions. The study team had some concern regarding the social desirability bias in which participants would be influenced by the expectations of their communities to give specific responses regarding MDA. To avoid this, the study team prefaced each interview with the goals of the research including reassurance that the participant’s honest feedback would help to improve their community’s health programs. The study team also conducted the interviews either in a closed office space or out in a field away from non-participants to allow for privacy and open discourse. The interviews took place immediately after the drugs were administered in both the trial and control arms.

Both the health work force key informant interview guide and the MDA recipient focus group interview guide included questions on socio-demographic information and open-ended questions including views on: pill burden; previous, non-integrated MDA campaigns; the occurrence and cause of non-compliance during MDA; and the time required for co-administered and non-co-administered MDA (S2 Text). The health work force key informant interview guide also included questions specific to the role the person played in the health work (S3 Text). To determine if any new or divergent responses occurred during an interview, interviewers used probing questions to explore the response and also used the new information to create follow-up questions for future interviews. Given the same two interviewers conducted all of the interviews, this approach led to eventual saturation in terms of new information provided.

### Data management and analysis

Interviewers recorded and transcribed verbatim into Afaan oromoo. The same interviewers that conducted the interviews translated them into English. The study team reviewed the transcribed interview data and flagged statements from participants that were significant, insightful, or frequently repeated. As Ethiopia has an established history of MDA but not of co-administration, certain codes surrounding MDA were previously identified while emerging codes were also important. Using NVIVO 12, the study team assigned deductive codes based on key themes, identified from both published literature and practical experience in Ethiopia, and added new codes based on insights that occurred during the review of the transcripts themselves. We used descriptive codes relating to the participant demographics (age, gender, etc.) and thematic codes relating to what was said by the participants. We then explored patterns in responses, linkages between the thematic and descriptive codes, and often repeated statements which required follow-up to avoid generalizations.

## Results

### Awareness creation and community sensitization about MDA

As is standard, the trial study team carried out community awareness campaigns prior to the MDA for both intervention and control groups. Dissemination of information took place through public gatherings, community leaders, health extension personnel, development armies, and other community members. The cooperation between health workers, village leaders, and community volunteers was cited as an excellent example of how to administer drugs successfully compared to previous MDAs. Participants stated that with previous MDAs this coordination was not common resulting in less than adequate social mobilization and preparation:

*“…There was a big meeting conducted; they provided us the information on that meeting*. *The health professionals had identified the pregnant women and given special care. Those who were not available at the time the tablets were given… are eager to get them. It was not like this in the previous times. They are serving us properly. People had benefited from that. I was feeling a disturbance in my stomach (nervousness); I got relief after I took it. When they give a tablet today, they recheck it the next day. To see whether there is a side effect or not*. *Because they were serving us just like this, the community is very happy”- (Female youths FGD)*

All participants mentioned the value of these community awareness events in ensuring village residents were aware of the MDA.

### House to house MDA implementation strategy compared to central point MDA strategy

During the study, we employed a house-to-house MDA strategy. Volunteers informed each village of the day and time that the study team would conduct the distribution. This differed from the previous MDA approach in this population, in which MDA was conducted at a central point in the village and community members had to attend within a given window to be treated.

The majority of participants said that the house-to-house MDA strategy used in the trial was preferable to the central point distribution strategy and enabled the majority of the community to receive the drugs. Participants were appreciative of the house-to-house delivery method. The early morning timing of drug delivery, team makeup, counseling and advice provided, and post-MDA follow-up that formed part of the RCT were all appreciated.

*“The drug administration was done in a good manner*. *It was not common for the district, health professionals, and the kebele leaders to collaborate in the way they are doing it now. They were serving each household in time. People are eagerly waiting for them [drug distributors]. They give the tablets/drugs today and revisit them the next day. It was not common before”. [FGD, Adult man, 48yrs]*

Using the previous central point strategy, it was reported that many members of the community had missed the MDA for a variety of reasons, including distance, being busy, a lack of available information and limited availability of health care staff.

Within the RCT, anyone who missed treatment had the chance to take it the next day thanks to the study follow-up visit.

“*Previously…*.*there was no follow-up*. *Follow-up [re-visit] and house-to-house service is done for the first time*. *In the current distribution*, *they are telling us that there will be no problem because we are following you closely*. *They never move to the next group*, *without checking up on the one already provided to*. *People are very happy about this and received the drug very well*” [FGD, Female Youth]

However, some study participants mentioned that the community members were unsure of the need for the follow-up. One health extension worker (HEW) stated:

“*They [community] are not familiar with the revisit [follow-up] after providing the drugs*. *They are suspicious of our revisit [follow-up]*. *They think that the drugs are harmful*. *We tell them that we are watching them if there is a side effect from the tablets*. *We tell them that we can give them assistance and take them to a health facility if there is any harm*” [KII, HEW].

Health workers also mentioned the effectiveness of the current MDA approach and its role in expanding coverage and reach as strengths:

“*The current MDA is very suitable*. *Because we are providing it by going to their residence*. *They are not willing to come to the place we want them to come…*. *Because we go to them early in the morning*, *we get everyone at home*. *At the time we used to gather them at one place in the previous MDA*, *only a few old men are coming*. *The majority of them are mothers and their children*. *This time we get everyone at home*” [KII, HEW]

### Perception towards pills burden during co-administration

The majority of study participants did not feel that taking a larger number of pills was more difficult compared to the previous separate MDAs. Many reported that they initially felt apprehensive but that the presence of supervisors at the time of drug administration, as well as provision of adequate information, advice, and counseling, made people feel comfortable taking the drugs.

“*It was very nice*. *Many people came together and gave us the tablet*. *Health extension workers*, *health professionals*, *those from district and zone were together in a team while providing the tablets*. *We got to know each other and then they told us the details of each tablet*. *That is how they provided it to us*. *The presence of many people (study team) while providing the tablet increases the acceptance*. *I wouldn’t even receive if I wasn’t convinced and understood the benefit to each drugs” [FGD*, *Adult woman*, *30 yrs]*

Some community members, particularly young women, expressed concerns that the taking so much of the drug at one time might lead to infertility. These concerns were allayed by a majority of community members receiving counseling and assistance from the staff responsible for administering drugs. An elder highlighted the absence of any issue related to the burden of pills in his village’s communities.

“*The number of pills did not affect anyone*. *People may be afraid of it but because the health professional know the way*, *there are no worries…… Because it is safe*, *the community is taking it*. *It has been more than 20 days since [drug distribution] began*, *I saw no single challenge so far*” [KII, Elder man, 60 yrs]

“*Nine tablets are not harder*. *I was afraid when taking it*, *but because I took it gradually in two rounds*, *it is not that hard*. *They were patient giving it to us*. *That is why we didn’t feel anything*” [FGD, Youth female]

The district NTD focal point stated that, in comparison to previous MDAs, more community members took part with the notable exception of some youth:

“*Some youth refused*. *Some of them politicized it*. *Some of them argue how and why to take drugs without getting sick and get diagnosed*.*”*. [KII, Health Worker, 37 yrs]

This hesitancy may have been linked with the recent introduction of the COVID-19 vaccine program which health workers noted had been met with hesitancy and suspicion among the youth in the communities. The introduction of a novel, multiple drug distribution strategy among the community stirred rumors that it was a replacement to COVID-19 vaccine for those who refused the injection. As the study continued and more community members received the drug, including religious and kebele leaders, the majority of youth eventually accepted the co-administered NTD medicine regimen.

Interviewees emphasized the importance of co-administration for saving time and preventing people being ‘missed’ during MDA:

“*I choose the nine tablets taken at once*. *Because re-visit (second MDA) can create a miss*, *as well a burden for a person delivering the drugs*” [KII, Aba Gada, 45 yrs]

*“Coming twice (for different MDAs) is just wasting time*. *If the nine tablets did not have any harm*, *it is better to give them at once*. *It solves the problems of drop out and missing”*. [KII, HDA, 45yrs]

### Overall perceptions of the integrated MDA compared to previous MDAs

As well as using a central point method, previous MDAs had relied heavily on community drug distributors rather than health care workers to conduct MDA. Some study participants reported that this strategy had resulted in concerns from the community that drugs were provided by volunteers.

“…*the volunteer did not get payment*, *but they need to get some money*. *It was difficult to identify who took drugs and not in previous MDA*. *In addition*, *there is a complaint on the side of the community*. *They complain about the MDA provided by farmers selected from the community*. *There are community members who say*, *how you dare you allow a farmer to deliver us a medicine in the previous MDA”*. [KII, Health Worker, 37 yrs]

Previously, MDA was carried out at various times of the year, such as during harvest, sowing, or cultivation depending on the timing that drugs were available. Even though our co-administered MDA occurred during harvest, the time [early morning distribution] and method [house-to-house distribution] were chosen to maximize participation. Communities in the area have a custom of staying at home until 10:00 AM before leaving for fieldwork, which made it easier to contact household members after breakfast.

Study participants advised administering MDA before or after harvest, when people are less busy, and during the dry season because transportation is also difficult during the rainy months. Participants in the study said that, with the exception of a small number of individuals who first displayed opposition, all community members, regardless of their gender, age, or religion, took part in the current MDA. Overall, all study participants acknowledged the MDA’s primary strategies, such as community awareness and sensitization, house-to-house distribution, early morning distribution, a second visit the next day, the team composition, and collaboration with local leaders as major factors in the high level of acceptance.

## Discussion

Co-administration of drugs for multiple NTDs during MDA has the potential to accelerate progress and save time of both providers and recipients. While several combinations of NTD drugs have been proven safe [2–6], there remain critical lessons to learn about how to implement this strategy and how it will be perceived by those who are actually involved in its implementation. MDA can be described transactionally by categorizing a demand side (households and community members) and a supply side (the health workforce) [10]. Our study demonstrates that co-administration, at least in this part of Ethiopia, is highly acceptable to both groups, with multiple perceived advantages over separate MDA delivery. In addition, our study provides insight into other aspects of optimizing MDA.

Central or fixed-point MDAs are used for many NTD elimination and control programmes. Previous studies have found that bringing the entire community together saves on both cost and time compared to house-to-house treatment, in particular in communities where houses are far apart and difficult to reach [11,12]. A downside of this approach is that it shifts much of the participation burden on to communities and may create barriers to access. The original intention of the coadministration safety RCT in Ethiopia was to use central point MDA in order to mirror the standard MDA implementation methodology as closely as possible. However, during the COVID-19 pandemic, the Ministry of Health required MDA distributors to move house-to-house to prevent large gatherings, based on WHO recommendations [13]. This model was still in place when our study took place.

Our respondents indicated that, even outside of the specific question of co-administration, a house-to-house approach is preferable. This model reduced barriers to participation, such as aligning work schedules with central point MDA schedules. In terms of co-administration, it also allowed for a more direct health education exchange with individual families to address any concerns related to this new MDA approach.

Some of our respondents described initial concerns about taking up to nine pills at one time. NTD programshave gone to great lengths in recent years to reduce the chance of choking [14]. Recent studies and policy documents have discussed a variety of possible causes of previous choking episodes, including pressure for compliance from the health work force, social pressure amongst beneficiaries, a lack of patient awareness and a lack of perceived right of refusal [14,15]. We created specific diagrams for the health work force to use during co-administration, encouraging a ‘two pills, swallow, pause, two pills…’ rhythm (S3). Such reference materials could be included in future larger scale co-administration campaigns. Given that as many as three MDAs are being folded into one co-administered campaign, national programs, NGDO partners, and donors should consider increasing the implementation time to allow for house-to-house visits with built in time for awareness creation and patient empowerment, hopefully utilizing costs saved from the combined MDA platform. Though not evaluated in the study, the use by adults of azithromycin oral suspension (in lieu of azithromycin tablets) could reduce the adult pill burden from nine tablets (four tablets of azithromycin, four tablets of ivermectin, and one tablet of albendazole) to five tablets (four tablets of ivermectin and one tablet of albendazole), though would require significant changes to manufacturing and supply chain processes. In line with existing guidance, however, oral solution should be considered for nervous participants of any age, to help increase community compliance and comfort [16].

As MDA was conducted as part of a trial, distribution teams consisted of the health development army, health extension workers and staff from the woreda and zonal level health departments, with support from community leaders. Previous studies have noted that new community-based medical interventions benefit from the participation of such respected individuals from the outset [17,18]. We noted that the involvement of members of the formal health system was highly valued and viewed as enhancing trust in the MDA compared to delivery solely reliant on the community health workforce.

There were some limitations to the study. The design of the randomized control trial in which this study was nested took place in a sub-set of a woreda and required intensive social mobilization, community sensitization, increased supervision and mandatory active and passive follow-up mechanisms. Standard mass drug administration campaigns may require the treatment of hundreds of districts and millions of people so the intensified supervisory parameters of the study may have created acceptability perceptions among the community which would not have occurred normally. It is also important to note that these districts have already received several rounds of MDA for both trachoma and LF and that a community which is new to MDA may have very different idea surrounding co-administration.

MDA campaigns require a significant time investment for the local health workforce, draw personnel away from other duties, and multiple stand-alone MDA days may therefore cause MDA fatigue within recipient communities. Studies conducted in other countries as well as within Ethiopia have demonstrated that MDA duties prevent volunteers from pursuing other employment and income generating activities [19]. Co-administration could be a way to significantly reduce the burden on the health workforce and was viewed positively by healthcare workers in the current study. It is important to note the importance of outlining roles and responsibilities of each health worker cadre as tasks may shift within an integrated model [20].

Co-administration could have significant programmatic impact, particularly in countries such as Ethiopia where large populations require MDA for multiple NTDs. Combining MDA could save money through the implementation of joint supply chains, health workforce training, drug administration and supervision, [21] and reduce the burden on communities. Our data suggest that with the correct implementation strategy such an approach is acceptable to both communities and staff and support widespread rollout of this approach.

## Supporting information

S1 Table Composition of Focus Groups and Key Informant Interviews. (DOCX)

S1 Text Position Descriptions. (DOCX)

S2 Text Health Extension Worker and Health Development Army Interview Guide. (DOCX)

S3 Text Community Member Focus Group Guide. (DOCX)
