# Supplementary material for: Perceptions and acceptability of co-administered albendazole, ivermectin and azithromycin mass drug administration, among the health workforce and recipient communities in Ethiopia
Source: PLoS Negl Trop Dis. 2023 Oct 2;17(10):e0011332. doi: 10.1371/journal.pntd.0011332 (PMC10569502; doi:10.1371/journal.pntd.0011332)
Supplement: S1 Table — (DOCX) [file pntd.0011332.s001.docx]

**Supplementary File S1: Compositions of focus groups and KII participants**

Key Informants:

| Zonal NTD Focal Person | 1 |
| --- | --- |
| District NTD focal person | 1 |
| Health Extension worker | 2 |
| Health Development Army | 3 |
| Kebele leader | 2 |
| Elder | 2 |
| Aba Gada | 2 |
| Community volunteer | 1 |

Focus Group: Male Adults:

| Code | MDA category | Kebele | Age | Educational status | Role | Marital status |
| --- | --- | --- | --- | --- | --- | --- |
| R2 | Control | Gurmicho | 50 | Grade 8 | Kebele Leader | Married |
| R3 | Control | Gurmicho | 58 | Grade 9 | Farmer | Married |
| R4 | Intervention | Alkaso | 66 | Grade 8 | Farmer | Married |
| R9 | Control | Gurmicho | 35 | Grade 3 | Farmer | Married |
| R7 | Control | Gurmicho | 35 | Grade 8 | Farmer | Married |
| R8 | Control | Gurmicho | 53 | Grade 4 | Farmer | Married |
| R1 | Intervention | Alkaso | 50 | Grade 7 | Farmer | Married |
| R5 | Intervention | Alkaso | 28 | Grade 10 | Farmer | Married |
| R6 | Intervention | Alkaso | 48 | Diploma | Farmer | Married |

Focus Group: Female Adults

| Code | MDA category | Kebele | Age | Educational status | Role | Marital status |
| --- | --- | --- | --- | --- | --- | --- |
| R1 | Control | Alkaso | 35 | Grade 12 | Farmer | Married |
| R2 | Control | Alkaso | 25 | Grade 8 | Farmer | Married |
| R3 | Control | Gurmicho | 25 | Grade 10+1 | Farmer | Married |
| R4 | Control | Gurmicho | 40 | Grade 2 | Farmer | Married |
| R5 | Intervention | Gurmicho | 30 | Grade 10+3 | Farmer | Married |
| R6 | Intervention | Gurmicho | 25 | Grade 8 | Farmer | Married |
| R7 | Intervention | Gurmicho | 26 | Grade 6 | Farmer | Married |
| R8 | Intervention | Alkaso | 30 | Grade 10 | Farmer | Married |

Male youths

| Code | MDA category | Kebele | Age | Education | Role | Marital status |
| --- | --- | --- | --- | --- | --- | --- |
| R1 | Control | Alkaso | 25 | Grade 7 | Farmer | Married |
| R2 | Intervention | Gurmicho | 23 | Diploma | Farmer | Single |
| R3 | Control | Gurmicho | 22 | Grade 6 | Farmer | Married |
| R4 | Control | Gurmicho | 21 | Grade 10 | Farmer | Single |
| R5 | Control | Alkaso | 37 | Grade 9 | Farmer | Married |
| R6 | Intervention | Gurmicho | 25 | Illiterate | Farmer | Married |
| R7 | Intervention | Alkaso | 22 | Grade 10 | Farmer | Married |
| R8 | Control | Gurmicho | 18 | Grade 7 | Farmer | Married |

Focus Group: Female youths

| Code | MDA category | Kebele | Age | Educational status | Role | Marital status |
| --- | --- | --- | --- | --- | --- | --- |
| R1 | Control | Alkaso | 35 | Grade 12 | Farmer | Married |
| R2 | Control | Alkaso | 25 | Grade 8 | Farmer | Married |
| R3 | Control | Gurmicho | 25 | Grade 10+1 | Farmer | Married |
| R4 | Control | Gurmicho | 40 | Grade 2 | Farmer | Married |
| R5 | Intervention | Gurmicho | 30 | Grade 10+3 | Farmer | Married |
| R6 | Intervention | Gurmicho | 25 | Grade 8 | Farmer | Married |
| R7 | Intervention | Gurmicho | 30 | Grade 6 | Farmer | Married |
| R8 | Intervention | Alkaso | 25 | Grade 10 | Farmer | Married |
