# Supplementary material for: Perceptions and acceptability of co-administered albendazole, ivermectin and azithromycin mass drug administration, among the health workforce and recipient communities in Ethiopia
Source: PLoS Negl Trop Dis. 2023 Oct 2;17(10):e0011332. doi: 10.1371/journal.pntd.0011332 (PMC10569502; doi:10.1371/journal.pntd.0011332)
Supplement: S1 Text — (DOCX) [file pntd.0011332.s002.docx]

**Supplementary File S1: Position Descriptions**

**Health Development Army:** “The Health Extension Program (HEP) launched in 2003, expanded basic health infrastructure and local human resources. In 2011, the government introduced the Health Development Army (HDA). HDA is a women-centered community movement inspired by military structures and discipline. Its special objective is to improve maternal health outcomes.”

**Health Extension Worker:** In Ethiopia, two Health Extension Workers (HEWs) are assigned per kebele, which is the lowest administrative unit of the government structure with an average of 1,000 households and approximately 5,000 people. HEWs provide services at their health post and in the community. To extend the reach and effectiveness of the HEWs, the Women’s Development Army (WDA) was organized in 2011. The WDA engages communities by organizing five or six neighboring households into teams, with each team selecting a WDA Volunteer from a model household (defined by adoption of healthy behaviors). At present, Ethiopia has approximately 40,000 HEWs and an estimated three million WDA Volunteers.

**Village Elders:**  **Village elders, known locally as "Jarsa Biyya", and respected by the community. They take part in social and cultural activities like conflict resolution. They represent community members on social issues and facilitate social activities like weddings and funeral ceremonies.**

**Aba Gada (community leader):** The Gada is a traditional system of governance of the Oromo people built off of community experience over generations which encompasses of all of the socio-political issues within a community . The leader of the Gada is ‘Aba Gada’ or father of the Gada.

**Muslim Religious Leader: These are persons who lead and teach Muslim religious scholars in a mosque. According to the tenants of the religion, there are different levels of leadership and teaching, such as sheiks, imams, and others.**

**Kebele Leader:** Politically nominated individual based within the kebele that leads on aspects of sub-woreda governance.

**Woreda NTD Focal Person:** Woreda NTD focal persons are based within the woreda health office and usually are the focal persons for multiple different health initiatives within their district.

**Zonal NTD Focal Person:** Zonal NTD focal person are based in the Zonal Health Department. They report to the Oromia Regional Health Bureau and often have multiple health initiatives to manage with their zone.
