# Supplementary material for: Perceptions and acceptability of co-administered albendazole, ivermectin and azithromycin mass drug administration, among the health workforce and recipient communities in Ethiopia
Source: PLoS Negl Trop Dis. 2023 Oct 2;17(10):e0011332. doi: 10.1371/journal.pntd.0011332 (PMC10569502; doi:10.1371/journal.pntd.0011332)
Supplement: S2 Text — (DOCX) [file pntd.0011332.s003.docx]

**Supplementary File**

**HEWs and HAD – KII- Interview Guide**

1. **Self-introduction;**

a. Age: ____________

b. Sex: ____________

c. Marital status: __________________

d. Educational status: _______________

e. Occupation (social responsibility): ______________________

f. Experience at the position: ___________________________

1. **Awareness of mass drug distribution**
2. Can you tell me about any recent mass drug distribution in this community so far you know? (**Probe further on:**
3. **When was the MDA conducted?**
4. **Why was it?**
5. **Process of mass drug distribution**
   1. **Who facilitated it?**
   2. *How was the community engagement work conducted?*
   3. *By whom was the distribution done?*
      - 1. **(Probe for :**
      1. *Pre distribution exercises, time of the day and feasibility strategy used)*
   4. **How was the community’s participation and acceptance, probe for?**
      1. *distribution exercises, time of the day and feasibility strategy used)*
         1. In your opinion did everyone in the community received the drugs? **(probe further on:**

- *If there are people or groups of people that don’t received/exempted, why was that?*
- *Could you please tell me any information you might have whether the missed individuals/groups were related to operational challenge, refusal by the individual/group or any other issue?*
- *Could you please tell me; if you know/heard of any justification/reason by individuals/groups for not participating in the MDA*
- *In your opinion what need be done to enable those who missed to receive the drugs?*

1. **Opinion about mass drug distribution that occurred this/past month**
   - - 1. Could you explain the arm of distribution in your community (triple co-administration -three drugs administered at ones or the standard MDA-two separate MDAs run in two weeks)
       2. Could you comment on the satisfaction of your community with the present way of drug distribution? **(Probe further on:**

- *What satisfied the community in the current MDA Approach?*
- *what was different in the current MDA approach that made it different from previous MDAs*
- *Was there any rumour/complaints; how did you heard about them; in your opinion is the rumour or complaint is shared by the community or is from some individuals.*
  - - 1. What suggestions does your community has, reiterate on the arm of the study of the interviewee, has for improving future distribution exercises (**probe further on**:
  - *Could you please tell us about the communities believes that need be improves; pill number (burden), strategy, timing, distributors)*

1. **Community factors**
2. Could you comment on the community’s perceptions towards the types of MDA, triple-co administration vs standard (**probe further on:** reported complaints on supply, sociocultural factors, religious factors….etc)
3. **MDA related Factors**
   - 1. What are the triple co-administration related challenges/concern that the community is raising?
        - Could you tell me, if there was/were effort(s) by the community to overcome/minimize challenges?
     2. How do community, family and/or close associate relate any complaint to the approach? (Probe further on

*the nature of the complaints [ most common compliant/duration and number*), process of MDA, drug related complaint, timing, etc

- Thank you for your, time and the information you provided; I am done with my question; I wonder if you have something different that you might have and you think would help; please tell me ?
