# Supplementary material for: Perceptions and acceptability of co-administered albendazole, ivermectin and azithromycin mass drug administration, among the health workforce and recipient communities in Ethiopia
Source: PLoS Negl Trop Dis. 2023 Oct 2;17(10):e0011332. doi: 10.1371/journal.pntd.0011332 (PMC10569502; doi:10.1371/journal.pntd.0011332)
Supplement: S3 Text — (DOCX) [file pntd.0011332.s004.docx]

**FOCUS GROUP DISCUSSION GUIDE –COMMUNITY VOLUNTEERS**

**FGD Category:** _________________ [***Adult Male, Adult Female, Youth Male and Youth Female***]

**FGD-Participant profile**

| Code | Age | Educational status | Occupation | Marital status | Social responsibility |
| --- | --- | --- | --- | --- | --- |
| P1 |  |  |  |  |  |
| P2 |  |  |  |  |  |
| P3 |  |  |  |  |  |
| P4 |  |  |  |  |  |
| P5 |  |  |  |  |  |
| P5 |  |  |  |  |  |
| P6 |  |  |  |  |  |
| P7 |  |  |  |  |  |
| P8 |  |  |  |  |  |
| P9 |  |  |  |  |  |

1. **Awareness of Mass drug Administration;** Could you please tell me,

Has there been any mass drug Administration (MDA) in this community recently? (**Probe further on:**

- - *How long ago was this done?*
  - *What was the purpose of the MDA*?

1. How was the communities’ perception of the community engagement/awareness creation exercise? **(probe further on:**
   - *Who did the community engagement on the MDA?*
   - *What methods of information dissemination methods used?*
   - *Adequacy of information provided?*
   - *Doesyour community have any preferred way of communication?*
2. **Process of the mass drug Administration,** Could you please tell me,
   - - 1. How was the MDA was done? **(Probe for:** *Pre distribution exercises, time of the day and feasibility strategy used)*

- *What is the communities’ feedback/concern on the convenience of the day and time for the MDA?*
- *is there a preferred time and day for the community? why does the community prefer the timing/date?*
  - - 1. Did the community believe that everyone in the community received the drugs? **(probe further on:**
- *If there are people or groups of people that don’t receive/are exempted, why do they do that?*
- *What is needed to be done to enable them to receive the drugs?*

1. **Opinion about drug distribution,** *could you please tell me*
   - - 1. Which arm of the distribution was your community (triple co-administration or the standard MDA)
       2. Were your community members satisfied with the present way of drug distribution? **(Probe further on:**

- *What is satisfying in the current MDA Approach? [Approach, time of distribution, facilitation of distribution, etc.]*
- *How does the current MDA approach differ from previous MDA*
  - - 1. What do your communities suggest to improve future distribution exercises (**probe further on**:
  - *Community engagement, pill number (burden), strategy, timing, distributors*

1. **Community factors**
2. What are the communities’ perceptions towards the types of MDA, triple-co administration vs standard (**probe further on:**
   - *The number of complaints, type/seriousness of complaints, logistical issues related to pre-during and post-distribution, socio-cultural, religious factors.*
3. **MDA related Factors**
   - 1. were there triple co-administration-only related challenges raised by the community members? Please tell me, in what sense’
   - *Number of complaints, type/seriousness of complaints, logistical issues related to pre-during and post-distribution, socio-cultural, and religious factors.*
4. Could you tell me how the community attributed the complaint to a specific MDA arm; triple or standard; **(Probe further on**
   - How does the community explain the difference between the arm,
   - How did they like future MDAs to be done

Why they preferred the approach?

1. Do you have anything more you would love to tell me?
